# Supplementary material for: Peruvian Amaranth (kiwicha) Accumulates Higher Levels of the Unsaturated Linoleic Acid
Source: Int J Mol Sci. 2023 Mar 25;24(7):6215. doi: 10.3390/ijms24076215 (PMC10093863; doi:10.3390/ijms24076215)
Supplement: Supplementary file 1 [file ijms-24-06215-s001.zip › ijms-2274301-supplementary.pdf]

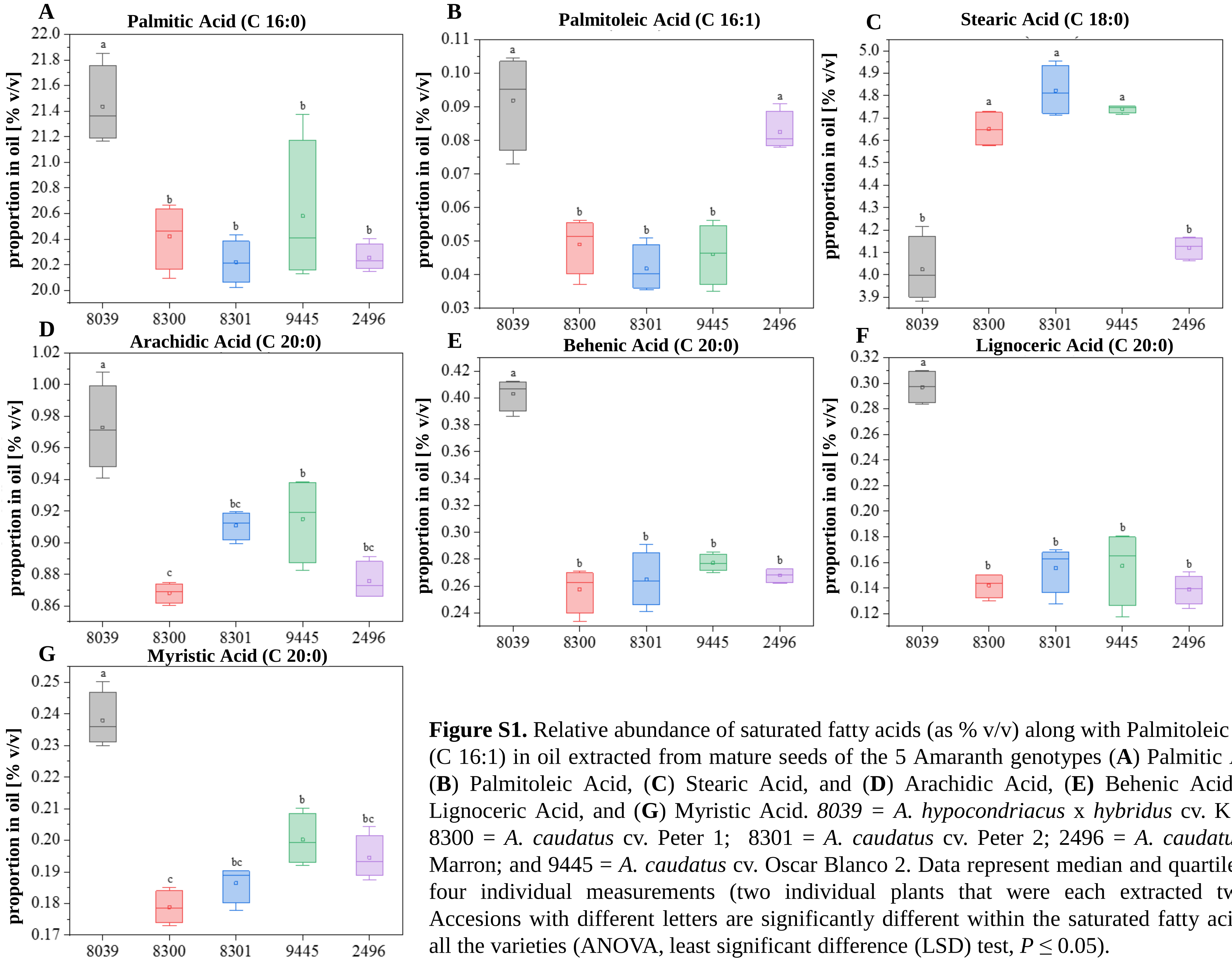

Figure S1. Relative abundance of saturated fatty acids (as % v/v) along with Palmitoleic Acid (C 16:1) in oil extracted from mature seeds of the 5 Amaranth genotypes (**A**) Palmitic Acid, (**B**) Palmitoleic Acid, (**C**) Stearic Acid, and (**D**) Arachidic Acid, (**E**) Behenic Acid, (**F**) Lignoceric Acid, and (**G**) Myristic Acid. *8039* = *A. hypocondriacus* x *hybridus* cv. K 432; 8300 = *A. caudatus* cv. Peter 1; 8301 = *A. caudatus* cv. Peter 2; 2496 = *A. caudatus* cv. Marron; and 9445 = *A. caudatus* cv. Oscar Blanco 2. Data represent median and quartiles for four individual measurements (two individual plants that were each extracted twice). Accesions with different letters are significantly different within the saturated fatty acid for all the varieties (ANOVA, least significant difference (LSD) test, $P \leq 0.05$).

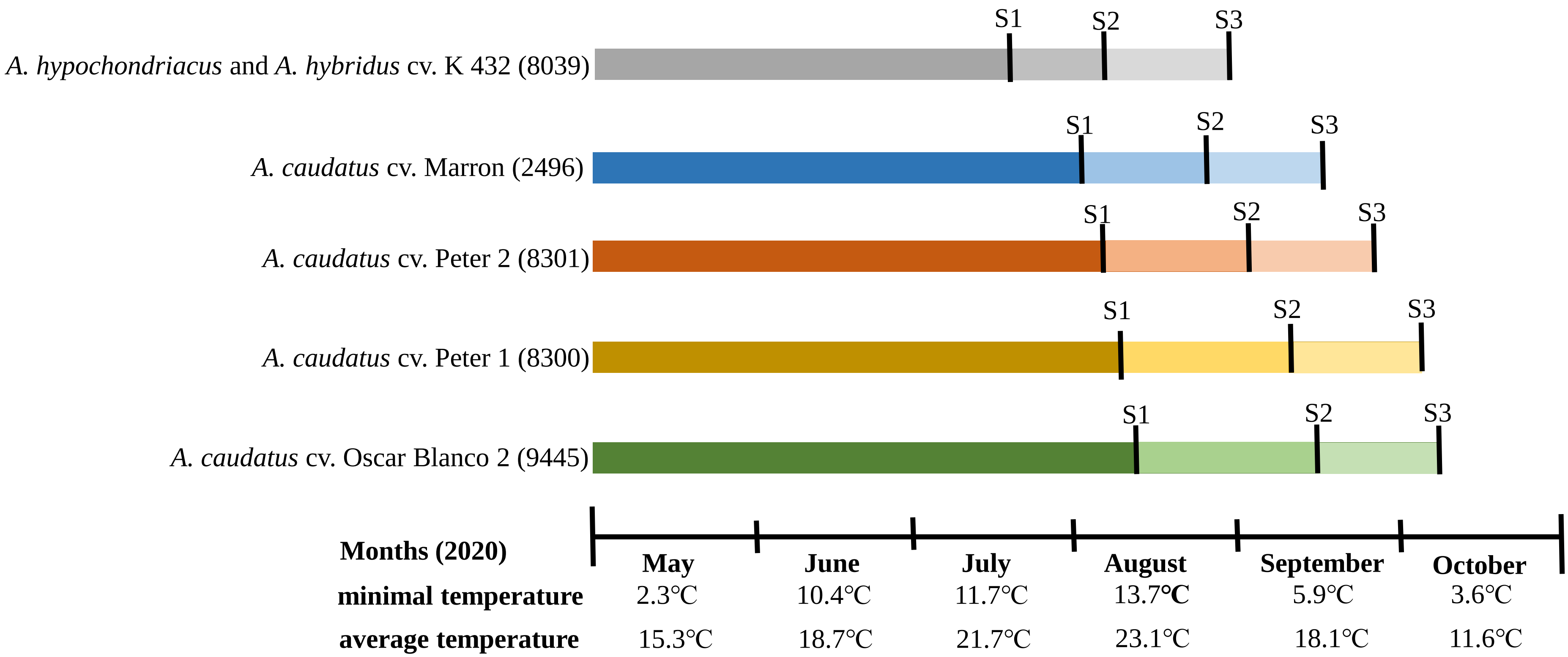

Figure S2. Temporal pattern of seed development in relation to average and minimal temperature during the growth of the five Amaranth genotypes evaluated under temperate environmental conditions in South-West Germany during the season 2020. S1: seed initiation; S2: milky state; and S3: maturity.

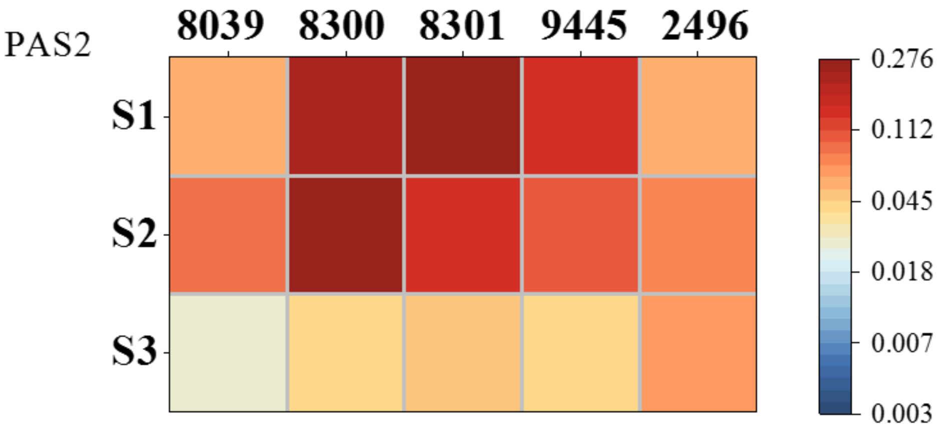

Figure S3. Steady-state transcript levels for Very-Long-Chain (3R)-3-Hydroxyacyl-CoA Dehydratase (PAS2). The plants were raised under temperate environmental conditions in South-West Germany during the season of 2020. S1 initiating seed development, S2 milky stage, and S3 seed maturity. 8039 = *A. hypocondriacus* x *hybridus* cv. K 432; 8300 = *A. caudatus* cv. Peter 1; 8301 = *A. caudatus* cv. Peter 2; 2496 = *A. caudatus* cv. Marron; and 9445 = *A. caudatus* cv. Oscar Blanco 2. Transcript levels were normalised against actin as internal standard. Values are means of three biological replicates each in technical triplicate. The colour scale gives the relative expression based on ΔC_t values.

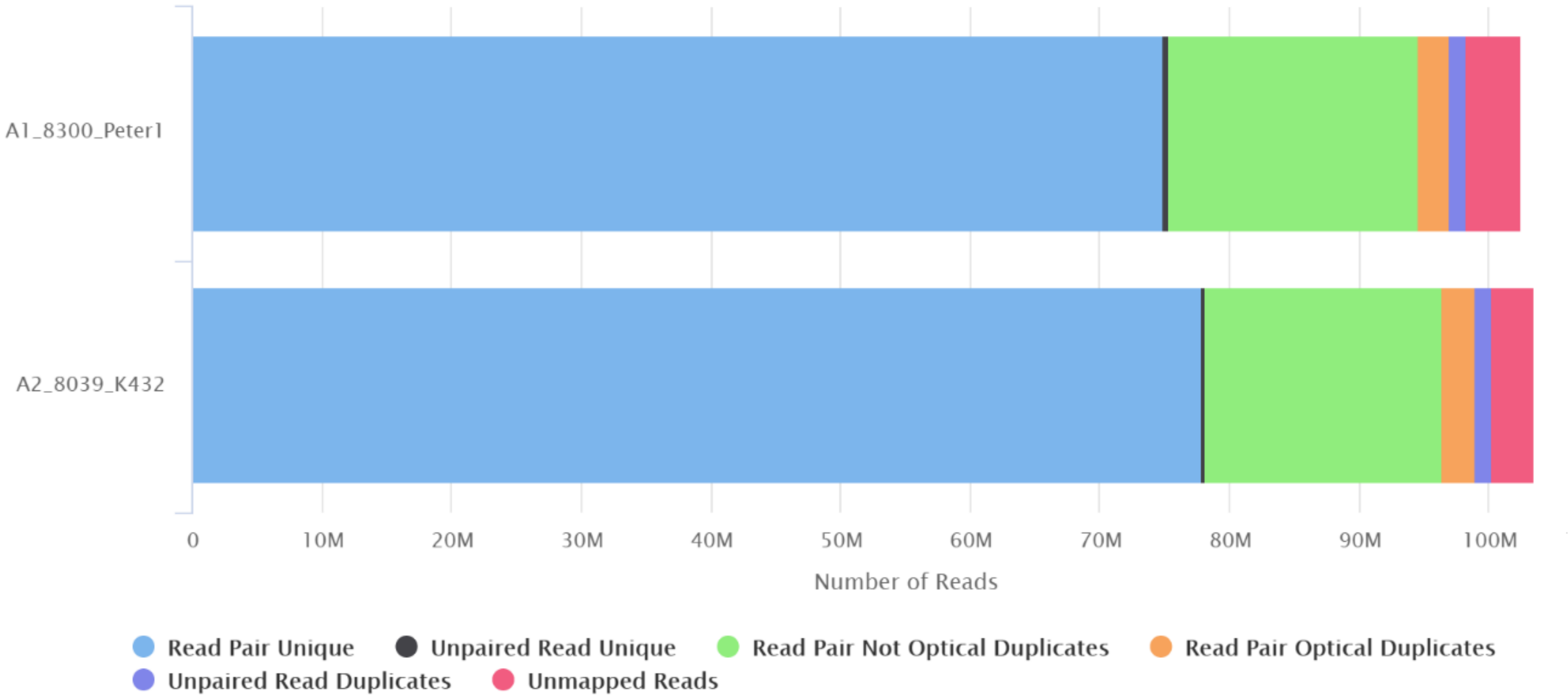

Figure S4: Graphical representation of variant statistics for the genomes of *A. caudatus* cv. Peter 1 (8300) and *A. hypocondriacus* x *hybridus* cv. K 432 (8039) genotypes. Heterozygous and homozygous variants were called from sequence alignments against the *A. hypochondriacus* PI 558499 reference genome (Lightfoot et al., 2017).

Table S1: Mean values of agro-morphological traits recorded from 5 Amaranth genotypes, their grouped overall mean values, least significant difference, and coefficient of variation as percentage (C.V%) conditions in South-West Germany during the season 2020.

ID KIT	Genotype's names	Days to flowering	Plant height (cm)	Inflorescence length (cm)	Grain yield (g/plant)	1000-seed weight (g)	Cross area of seed (mm^2)
9445	*A. caudatus* cv. Oscar Blanco 2	98.33 e	242.00 c	66.00 a	3.69 d	0.83 d	1.38 b
2496	*A. caudatus* cv. Marron	89.00 d	241.50 c	81.00 c	2.10 d	0.62 c	1.11 a
8300	*A. caudatus* cv. Peter 1	96.33 b	286.50 b	97.50 b	10.70 b	0.65 b	1.04 a
8301	*A. caudatus* cv. Peter 2	93.33 c	279.50 b	93.50 b	7.77 c	0.63 c	1.37 b
8039	*A. hypocondriacus* x *hybridus* cv. K 432	79.00 a	109.50 a	60.00 a	17.27 a	0.56 a	0.93 a
	Total mean	91.20	238.00	85.00	9.35	0.66	1.24
	L.S.D (5%)	1.45	14.47	8.13	2.36	0.02	0.16
	C.V%	0.84	3.22	5.08	13.42	1.926	7.04

Data were analysed by an F-test. LSD: least significant differences within the trait for all the varieties ($P \leq 0.05$). Values followed by the same letters are not significantly different at $P \leq 0.05$.

Table S2: Analysis of variance of six agro-morphological traits recorded from 5 Amaranth genotypes evaluated under temperate environmental conditions in South-West Germany during the season 2020.

Source	degree of freedom	Days to flowering	Plant height	Inflorencence length	Grain yield	Cross seed area	1000-seed weight
Genotypes	4	176.60****	15274.66****	964.50****	121.73****	0.12***	0.02****
Replication	2	20.60	658.40	442.40	17.50	0.09	0.00
Error	14	0.60	59.06	1.5780	74.05	0.00	0.00

*** and **** Significant at 0.001, and 0.0001 probability levels, respectively. ns= non-significant.

Table S3: Temperature and rainfall measured in Karlsruhe during the summer season 2020.

Month	Average temperature	Highest temperature	Lowest temperature	Rain
March	8.34 ℃	19.27 ℃	-1.35 ℃	53.6 mm
April	14.36 ℃	25.05 ℃	-0.67 ℃	6.7 mm
May	15.31 ℃	26.27 ℃	2.32 ℃	43.2 mm
June	18.75 ℃	28.98 ℃	10.42 ℃	74.7 mm
July	21.70 ℃	36.14 ℃	11.75 ℃	22.4 mm
August	23.06 ℃	35.72 ℃	13.75 ℃	50.5 mm
September	18.10 ℃	31.42 ℃	5.95 ℃	36.2 mm
October	11.61 ℃	22.43 ℃	3.65 ℃	47.8 mm
November	7.65 ℃	22.31 ℃	-3.05 ℃	21.3 mm
December	5.27 ℃	15.49 ℃	-0.62 ℃	79.9 mm

Table S4: Analysis of variance of ten fatty acid compositions measured in the seed oil of 5 Amaranth genotypes evaluated under temperate environmental conditions in South-West Germany during the season 2020.

	d.f	C14:0	C16:0	C16:1	C18:0	C18:1	C18:2	C18:3	C20:0	C22:0	C24:0
Genotype	4	0.0020^{****}	0.9947^{***}	0.0021^{****}	0.5507^{****}	8.2266^{***}	8.5334^{**}	0.0577^{***}	0.0069^{****}	0.0150^{****}	0.0179^{****}
Replication	3	0.0000^{ns}	0.1674^{ns}	0.0001^{ns}	0.0039^{ns}	0.1232^{ns}	0.1250^{ns}	0.0001^{ns}	0.0002^{ns}	0.0001^{ns}	0.0003^{ns}
Total error	12	0.0000	0.0853	0.0001	0.0097	0.8367	1.2891	0.0006	0.0004	0.0002	0.0003
Mean		0.19	20.58	0.06	4.47	20.76	51.73	0.81	0.91	0.29	0.18
CV%		3.48	1.41	14.61	2.20	4.41	2.19	3.05	2.20	4.86	10.14

, * and **** Significant at 0.01, 0.001, and 0.0001 probability levels, respectively. ns= non-significant; d.f = degrees of freedom; CV = Coefficient of variation.

Table S5: Variant statistics of *A. caudatus* cv. Peter 1 (8300) and *A. hypocondriacus* x *hybridus* cv. K 432 (80399 genotypes. Heterozygous and homozygous variants were called from sequence alignments against the *A. hypochondriacus* PI 558499 reference genome [78] and expressed as percentage rates.

variant statistics	*A. caudatus* cv. Peter 1 (8300)	*A. hypocondriacus* x *hybridus* cv. K 432 (8039)
total variants	3,703,021	1,592,370
SNPs	3,071,634	1,243,281
insertions	270,883	147,099
deletions	353,977	199,155
InDels	488	550
heterozygous variants	517,057	836,128
homozygous variants	3,185,964	756,242
het/hom ratio	0.16	1.11

Table S7: List of oligonucleotide primers used for RT-qPCR analysis in this study.

Name	Sequence (5'-3')	GenBank accession no. (phytozome database)	product size (bp)
Ah.ACT-F	CGTGACCTGACTGATTACCTTA	AH008914	178
Ah.ACT-R	GCTCGTAGTTCTTCTCAATGGC		
Ah.FAD2-F	AGGGTCTAGCTTGGGTGGTT	AH010897	139
Ah.FAD2-2-R	CGATGAGTTGTAGTGGGGCA		
Ah.FAD7-F	TGTCATGTGGTTGGACTTGG	AH016385	110
Ah.FAD7-R	GTGTTGTAAGCCCTCCTCGG		
Ah.FAD-D6-F	TTCGTGCTCTCGAGTTTCGT	AH009625	92
Ah.FAD-D6-R	TCAGGCTGACCGACATACAC		
Ah.PSE1-F	CGCCATTTCCGAGATCCGAG	AH008435	125
Ah.PSE1-R	AGAAGAAAACCCGACCCGTT		
Ah.PAS2-F	GCTTTGGATGGGCACAAGTT	AH003652	93
Ah.PAS2-R	GAAGAGGCTTCTCAACGGCG		
Ah.Lipoxygenase-F	AGTACGATGTTCCAGCCGTC	AH004925	83
Ah.Lipoxygenae-R	TCCTGCTAAAGCCTGTCGTG		
Ah.AOS-F	CCCGAGCGTCCACAATAAAC	AH000843	129
Ah.AOS-R	GCACCCAAAGGCGAATAACC		
Ah.AOC-F	AGGGTGATTTGCCTAAAGAGC	AH022900	88
Ah.AOC-R	GGGAGAGTGGCCTTAGCATC		
Ah.JAZ1-F	CAGTGGGGGAGAAGCAAGTT	AH014315	107
Ah.JAZ1-R	GGTCATTTGGGGGATCGGAG		
Ah.ICE1-F	ACTTCCGAACCTGCCATCTC	AH017386	120
Ah.ICE1-R	TGGATGTTGACGGCTCTTCC		
Ah.CBF4-F	CGGCTCGAGCTCATGATGTA	AH012345	133
Ah.CBF4-R	GCAGCAGCACGCTGAATATC		
Ah.Xero2-F	GGTGCGAGATCTTCTGCGA	AH021067	119
Ah.Xero2-R	GTGGAGTGGAGGGAGAAATTG		
Ah.COR1-F	GGAATCCAGTACAGCACACG	AH005786	137
Ah.COR1-R	TCCCTCCGGTACCTATGACA		
